# Immunoglobulin G (IgG) attenuates neuroinflammation and improves neurobehavioral recovery after cervical spinal cord injury

**DOI:** 10.1186/1742-2094-9-224

**Published:** 2012-09-21

**Authors:** Dung Hoang Nguyen, Newton Cho, Kajana Satkunendrarajah, James W Austin, Jian Wang, Michael G Fehlings

**Affiliations:** 1 Institute of Medical Science, Faculty of Medicine, University of Toronto, Toronto, Canada; 2 Division of Genetics and Development, Toronto Western Research Institute, Toronto, Canada; 3 Krembil Neuroscience Center, University Health Network, Toronto, Canada; 4 Division of Neurosurgery, University of Toronto, Toronto, Canada; 5 University of Toronto Neuroscience Program, McL 12-407 399 Bathurst Street, Toronto, ON, M5T 2S8, Canada

**Keywords:** Spinal cord injury, Inflammation, Immuno-modulatory, Immunoglobulin G, Functional recovery

## Abstract

**Background:**

Evidence suggests that the inflammatory events in the acute phase of spinal cord injury (SCI) exacerbate the initial trauma to the cord leading to poor functional recovery. As a result, minimizing the detrimental aspects of the inflammatory response after SCI is a promising treatment strategy. In this regard, immunoglobulin G (IgG) from pooled human serum is a promising treatment candidate. Due to its putative, though poorly characterized immuno-modulatory effects, IgG has been used clinically to treat neuroinflammatory disorders such as Guillain-Barré syndrome, but its effects in neurotrauma remain largely unexplored.

**Methods:**

This study examines the potential neuroprotective effects of IgG in a well-characterized cervical model of SCI. Female Wistar rats were subject to moderate-severe clip compression injury at the C7-T1 level. IgG (0.4 g/kg) or saline was injected intravenously to randomly selected animals at 15 min post SCI. At several time points post SCI, biochemical assays, histology and immunohistochemistry analyses, and neurobehavioral assessments were used to examine the neuroprotective effects of IgG at the molecular, cellular, and neurobehavioral levels.

**Results:**

We found that intravenous treatment of IgG following acute clip-compression SCI at C7-T1 significantly reduced two important inflammatory cytokines: interleukin (IL)-1β and IL-6. This early reduction in pro-inflammatory signaling was associated with significant reductions in neutrophils in the spinal cord and reductions in the expression of myeloperoxidase and matrix metalloproteinase-9 in the injured spinal cord at 24 h after SCI. These beneficial effects of IgG were associated with enhanced tissue preservation, improved neurobehavioral recovery as measured by the BBB and inclined plane tests, and enhanced electrophysiological evidence of central axonal conduction as determined by motor-evoked potentials.

**Conclusion:**

The findings from this study indicate that IgG is a novel immuno-modulatory therapy which shows promise as a potential treatment for SCI.

## Introduction

Spinal cord injury (SCI) consists of two defined injury processes termed the ‘primary’ and ‘secondary’ injury [1-3]. The primary injury is caused by mechanical trauma to the spinal cord. The secondary injury involves a cascade of cellular and molecular events that leads to the destruction of spinal tissue integrity beyond the site of injury and results in sensory and motor deficits [2,4-6]. While the initial mechanical trauma causes immediate tissue destruction at the injury site, a myriad of neurotoxic substances during the secondary injury event destruct tissue in the penumbra region in a delayed and progressive fashion.

Neuroinflammation is implicated in orchestrating the secondary injury cascade after SCI [7,8]. Neutrophils, microglia, and macrophages can exacerbate SCI with their cellular products including pro-inflammatory cytokines, matrix metalloproteinases (MMPs), and reactive oxygen radicals. Many different pharmacological agents have been examined for their efficacy in attenuating inflammation-mediated damage after SCI, including methylprednisolone [9-11], minocycline [12-14], anti-CD11d/CD18 antibodies [15-17], neutrophil elastase inhibitor [18], secretory leukocyte protease inhibitor [19], and depletion of peripheral macrophages [20].

While a large number of immuno-modulatory agents have been examined at the preclinical stage, few studies have shown robust enough effects for these agents to be considered for clinical translation [21]. Only methylprednisolone has demonstrated some level of efficacy in improving patients’ functional recovery in Phase III clinical trials [22]. However, the effectiveness of methylprednisolone treatment is modest and is associated with increased susceptibility to infections and wound-related complications in SCI patients [23,24]. Thus there is an impetus to find a safe and effective therapy that attenuates inflammation-mediated damage and improves patients’ functional recovery after SCI.

Immunoglobulin G (IgG) is a potential treatment candidate. Intravenous use of IgG exhibits many immuno-modulatory properties and has been used clinically to treat several autoimmune diseases [25]. Currently, IgG is being investigated as an immuno-modulatory therapy in many neurological diseases that have a similar pathobiology to SCI, such as multiple sclerosis and stroke [26,27]. With the exception of one preliminary report describing how intraperitoneal injection of IgG immediately after SCI reduced myeloperoxidase activity in the injured spinal cord, the neuroprotective effects and the immuno-modulatory mechanisms of IgG in neurotrauma are currently unclear [28].

In this study, we examined a potential immuno-modulatory mechanism of IgG in the context of SCI and carried out a detailed characterization of IgG’s neuroprotective properties at the molecular, cellular, and neurobehavioral levels. We hypothesized that IgG treatment after SCI would attenuate inflammation-mediated damage and improve functional recovery. We provide novel evidence that IgG attenuates post-injury rises in interleukin (IL)-1β and IL-6, reduces the extent of neutrophil invasion and post-traumatic levels of MMP-9 and myeloperoxidase, is associated with perilesional tissue sparing, and promotes neurobehavioral and electrophysiological evidence of functional recovery.

## Material and methods

### Experimental and control groups

This study used 135 female Wistar rats (250 to 300 g) from Charles River and followed the animal-use protocol approved by the Animal Use Committee (AUC) of the University Health Network (#979) for all procedures. All rats underwent a C7-T2 laminectomy and were then randomly and blindly assigned to three groups (sham, saline, and IgG). A 35-g modified aneurysm clip was applied extradurally to animals in the saline and IgG groups at the level C7-T1 for 60 s to cause a moderate to severe spinal cord injury [29,30]. The spinal cords of sham animals were not injured. Animals were given 1 mL of buprenorphine (0.05 mg/kg) and 5 mL of saline immediately and subsequently every 12 h for 3 days after surgery.

IgG (I4506) from pooled human serum was purchased from Sigma Aldrich. After dissolving 500 mg of IgG in 4 mL of sterile saline solution (0.9% NaCl, Baxter), a single bolus of IgG at a dosage of 0.4 g/kg was injected intravenously through the tail-vein at 15 min after SCI. Control animals were injected intravenously through the tail-vein with 1 mL of sterile saline (0.9% NaCl). The dosage of 0.4 g/kg was chosen based on the reported dosage by Gok *et al.* and based on the clinical dosages that are currently used to treat Guillain-Barré syndrome and other autoimmune disorders [25,28]. There was no noticeable difference in the general behavior and appearance of animals that received either IgG or saline. There was also no difference in mortality rate between saline and IgG-treated animals. All assessors were blinded to the treatment groups throughout the study.

### Biochemical analyses

Following SCI, animals were sacrificed at 4 h for multiplex enzyme-linked immunosorbant assays (ELISA) and at 24 h for western blot and myeloperoxidase (MPO) activity analyses. Each animal was overdosed with pentobarbital and perfused with 180 mL of iced-cold saline (0.9% NaCl). The spinal cord was isolated in ice-cold Ringer’s solution and the meninges were removed. A 0.5 cm length of the spinal cord centered at the injury epicenter was dissected, immediately frozen with liquid nitrogen and crushed with a frozen mortar and pestle. The tissue was then stored at −80°C until use.

#### Myeloperoxidase activity assay

Myeloperoxidase (MPO) activity was determined using a MPO fluorometric kit available from Assay Designs (Enzo Life Sciences) according to manufacturer instructions. A total of 21 animals were used in this experiment (four sham, nine saline-treated, and eight IgG-treated animals). Briefly, cellular membranes were disrupted and blood was removed by homogenizing spinal cord tissue in the provided homogenization buffer (without detergent) containing 10 mM N-Ethylmaleimide. The samples were then centrifuged at 4°C at 12,000 g for 20 min, and the supernatant was removed. MPO was released from granules in pelleted material by homogenizing in solubilization buffer containing 0.5% of the detergent hexadecyltrimethylammonium (HTA-Br) (w/v) and also by exposing the mixture to two freeze/thaw cycles. The samples were then centrifuged at 8,000 g for 20 min at 4°C. The resultant supernatants were used in the assay. A Perkin-Elmer plate reader measured the fluorescence intensity, with excitation wavelength at 530 nm and emission wavelength at 590 nm. A calibration curve run concurrently with the samples was used to determine the MPO activity from the measured relative fluorescence intensity (RFU).

#### Western blot

A total of 21 animals were also used in this experiment (four sham, nine saline-treated, and eight IgG-treated animals). Spinal cord tissue was solubilized in 400 μL of RIPA buffer (25 mM Tris–HCl pH 7.6, 150 mM NaCl, 1% NP-40, 1% sodium deoxycholate, 0.1% SDS; Thermo Fisher) containing a cocktail of phosphatase and protease inhibitors. Protein concentration of each sample was measured using the Modified Lowry method [31,32]. All western blot reagents were purchased from Bio-Rad unless otherwise stated. An equal amount of protein (20 μg of total protein) per sample was separated using a 12% SDS-PAGE gel. Following electrophoresis, the gel was transferred onto a nitrocellulose membrane overnight. The membrane was then washed in 0.2% tween tris buffered saline (TBS-T) and blocked for 1 h in TBS-T + 5% milk. Primary antibodies were incubated overnight at 4°C, and the secondary antibody incubation step was accomplished at room temperature for 1 h (Table 1 lists the primary and secondary antibodies used for western blot analysis). The membrane was washed with TBS-T three times for 5 min each time in between each antibody incubation step. Enhanced ChemiLuminescence (ECL; Perkin Elmer) substrate for horseradish peroxidase (HRP) was used to detect the signal. Densitometry analysis was accomplished using Quantity One software (Biorad). 

**Table 1:** List of primary and secondary antibodies used for western blot analysis

**Antibody**	**Host**	**Company**	**Dilution**	**Duration**
Anti-rat β-actin	Mouse	Chemicon (MAB1501R)	1:500	Overnight 4°C
Anti-rat matrix metalloproteinase (MMP)-9	Rabbit	Chemicon (AB19016)	1:1,000	Overnight 4°C
Anti-rabbit IgG horseradish peroxidase (HRP)	Goat	Jackson Lab (11-035-144)	1:5,000	1 h RT
Anti-mouse IgG horseradish peroxidase (HRP)	Goat	Sigma (A-3682)	1:2,000	1 h RT

Antibody concentrations and incubation conditions are listed.

#### Enzyme-Linked Immunosorbant Assay (ELISA) analysis

Spinal cord homogenates of 19 animals (four sham, seven saline-treated, and eight IgG-treated animals) were generated using the same method described for the western blot procedure. Inflammatory cytokine (TNF-α, IL-1β, and IL-6) and chemokine (CINC-1 and MCP-1) levels in spinal cord homogenates were determined using multiplex ELISA. The ELISA procedure utilized was that of Eve Technologies (Calgary, AB, Canada) using plates commercially available from Millipore.

### Histological and immunohistochemical analysis

Animals were sacrificed at 24 h after SCI for immunohistochemical analysis and at 6 weeks after SCI for histological analysis. Each animal was overdosed with pentobarbital and then perfused with 60 mL of ice-cold phosphate-buffered saline (PBS) and 180 mL of para-formaldehyde (4% w/v in PBS, pH 7.4). The spinal cord of each animal was dissected and post-fixed overnight with 10% sucrose 4% para-formaldehyde PBS solution. The spinal cord was then cryo-protected in 20% sucrose PBS solution. The spinal cord tissue (1.0 cm centering on the injury epicenter) was embedded in tissue-tek and stored at −80°C. Spinal cords embedded into tissue-tek were cryosectioned at 20 μm thickness. Tissue sections were mounted on glass slides and stored at −80°C.

#### Cellular localization and tissue distribution of IgG at 24 h after SCI

Tissue sections from nine animals (three sham, three saline-treated, and three IgG-treated animals) were incubated in 1% SDS in PBS for antigen-retrieval and washed in between staining steps at least three times for at least 10 min each. Primary antibodies were incubated at 4°C overnight, and secondary antibodies were incubated at room temperature for 1 hour. Secondary antibody alone (no primary antibody) served as negative control. The same exposure and detector settings were used to acquire all images. Table 2 lists the primary and secondary antibodies used for the immunohistochemistry analyses.

**Table 2:** List of primary and secondary antibodies used for immunohistochemistry

**Antibody**	**Host**	**Company**	**Dilution**	**Duration**
Anti-rat ionized calcium binding adapter molecule (Iba)-1	Rabbit	Wako (019–19741)	1:300	Overnight 4°C
Anti-rat endothelial cell antibody (RECA-1)	Mouse	Serotec (MCA970R)	1:100	Overnight 4°C
Anti-human IgG-TRITC	Goat	Chemicon (AP504R)	1:300	1 h RT
Anti-rabbit IgG AlexaFluor 488	Goat	Invitrogen (A11034)	1:300	1 h RT
Anti-mouse IgG AlexaFluor 488	Goat	Invitrogen (A11001)	1:300	1 h RT

Antibody concentrations and incubation conditions are listed.

#### Stereological quantification of neutrophil infiltration

Tissue sections from 16 animals (four sham, seven saline-treated, and five IgG-treated animals) were stained following a similar protocol as above. Tissue sections were systematically sampled at every 240 μm over a distance of 3,000 μm (total of 14 tissue sections), which were centered at the lesion epicenter in each animal. Neutrophils were stained with anti-PMN (polymorphonuclear cells; Cedarlane Labs) primary antibodies and probed with anti-rabbit AlexaFluor 488 secondary antibodies. Neutrophils were quantified stereologically using the StereoInvestigator Software. Every cell that was positively stained with anti-PMN antibodies was cross-checked with a fluorescence nuclear stain signal, DAPI (4′, 6-diamidino-2-phenylindole) ensuring that every counted cell had a nucleus.

#### Luxol-fast blue/haematoxylin-eosin staining for histological analysis

At 6 weeks following injury, spinal cord tissue sections from 18 animals (four sham, seven saline-treated, and seven IgG-treated animals) were systematically sampled at every 120 μm over a distance of 4,000 μm (34 tissue sections per animal). Tissue sections were stained with luxol-fast blue (LFB) overnight at 56°C. Haematoxylin was used to stain nuclei and eosin was used as a counter-stain. After staining, tissue sections at which the injury epicenter localized were selected using the following criteria: (1) the largest area of scar and cavity relative to other tissue sections of the same animal; and (2) the smallest area of white matter and/or grey matter. After the injury epicenter center was located, other tissue sections were selected with respect to the injury epicenter. Tissue sections were analyzed using the Cavalieri Probe (StereoInvestigator software), which allowed the experimenter to manually measure the area of interest on each tissue section.

The following criteria ensured consistency during histological analysis: (1) cavity and scar area consisted of any area on the tissue section that was surrounded by scar tissue or appeared to be devoid of tissue; (2) white matter area consisted of any tissue region that appeared blue due to LFB staining; (3) grey matter consisted of any tissue region that appeared red due to eosin staining; and (4) preserved tissue area was the sum of the grey matter and white matter area. Scar tissue comprised any tissue area that had a fibrous and inconsistent tissue matrix. The percentage of scar and cavity area was calculated using the following formula:

(1) $$\%\;of\;scar\;and\;cavity\;of\;tissue\;\sec tion\;Y=\frac{area\;of\;scar\;and\;cavity\;of\;tissue\;section\;Y}{total\;area\;of\;section\;Y}$$

The percentage of preserved tissue was calculated using the following formula:

(2) $$\%\;of\;preserved\;tissue\;of\;\sec tion\;X=\frac{area\;of\;white\;and\;grey\;matter\;of\;tissue\;section\;X}{total\;area\;of\;section\;X}$$

### Assessment of functional recovery

#### Neurobehavioral assessment

A total of 31 animals (four sham, 13 saline, and 14 IgG-treated animals) were used for the neurobehavioral assessment and electrophysiological recordings. We assessed functional recovery using the inclined plane and the Basso Beattie Bresnahan (BBB) tests. The inclined plane test is an integrated neurobehavioral test that evaluates forelimb, hind-limb, and trunk control. The inclined plane test was originally described by Rivlin and Tator [33], and has been validated as a sensitive measure of injury at C7-T1 [30]. Performance on the inclined plane test correlates with the number of remaining axons belonging to non-pyramidal tracts including the rubrospinal, vestibulospinal, and raphespinal tracts [30,34]. The BBB test was used to make weekly assessments of the animals’ hind-limb function [35]. Two blinded observers evaluate the animal’s hind-limb function for 4 consecutive minutes and assign a score based on a 21-point locomotor rating system.

#### Electrophysiology

Motor-evoked potentials (MEPs) indicate descending motor pathway function, and the validity of this technique in animal models of SCI has been well demonstrated [36]. At 6 weeks after SCI, MEPs were recorded from 17 randomly selected animals (three sham, seven saline-treated, and seven IgG-treated animals), which had previously been used in the neurobehavioral assessment, under isofluorane anesthesia. Two stainless-steel needle electrodes were inserted into the tibialis for recording EMG signals. After removing inter-laminar ligaments between C1, C2, and C3, two pairs of 1.0 mm ball recording electrodes were positioned extradurally over the spinal cord, under the vertebral arch of C3. A constant current stimulus of 0.1 ms in duration and 2.0 mA in intensity was applied at a rate of 0.13 Hz to the dorsum at either C1 or C3. At a bandwidth of 10 to 3,000 Hz, a total of 500 MEPs were averaged and replicated using an electrodiagnostic device (Dantac Keypoint Portable, Denmark). Spinal axonal conduction velocity was calculated by dividing the distance between the stimulating electrodes by the difference between the peak latencies of distal and proximal stimuli.

### Statistical analysis

All statistical analyses were completed using SigmaStat Software. One-way analysis of variance (ANOVA) followed by a Bonferroni post-hoc test was used to determine the difference between treatments with regard to the biochemical, immunohistochemical, molecular and electrophysiology analyses. A two-way ANOVA followed by Bonferroni post-hoc test was used to determine significant differences between treatments in BBB and inclined plane tests over several time points. Since MMP-9 expression was not detectable in the sham group, the Student *t*-test was used to test the difference between saline and IgG-treated animals. All data are reported as mean ± SEM. Differences were considered significant at *P* <0.05.

## Results

### Systemically delivered IgG enters the injured spinal cord and co-localizes with astrocytes

**Figure 1:**
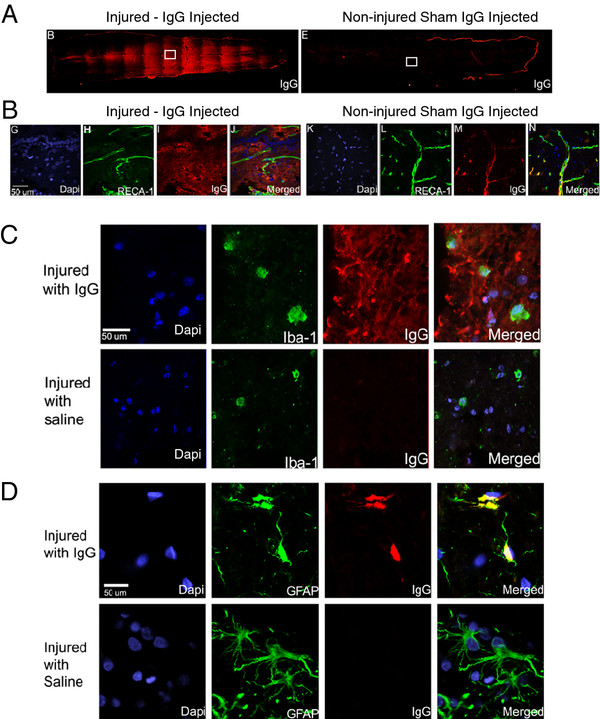
**IgG crosses the blood spinal cord barrier and associates with astrocytes.** (**A**) Representative fluorescence images showing the presence of IgG in the spinal cords of an injured rat (left) and a non-injured rat (right). (**B**) Vessels were stained with RECA-1 (green) and IgG was labeled with a human specific IgG secondary antibody (red). Confocal images demonstrate IgG was able to cross the blood-spinal cord barrier in injured animals (left panel) but not in non-injured animals (right panel). (**C**) Representative confocal images of microglia/macrophages (Iba-1; green) and IgG (red). IgG was observed in the parenchyma surrounding cells marked by Iba-1 and DAPI (blue). Although IgG was in the vicinity of Iba-1 positive cells, co-localization between IgG and Iba-1 was not observed. (**D**) Representative confocal images of astrocytes (GFAP; green) and IgG (red). IgG was observed in the parenchyma surrounding and in the cell soma of GFAP positive astrocytes. The co-localization of IgG and astrocytes suggests potential interaction between IgG and astrocytes. Note that an IgG-positive signal was not observed in the spinal cord of rats injected with saline. Confocal images (**B**-**D**) were taken from the boxed area, and scale bars represent 50 μm in length.

Fluorescence microscopy was used to show that IgG was present in the spinal cord of the injured rat 24 h following injury (Figure 1A). IgG (shown in red) could be seen caudal and rostral to the injury site and in the white and grey matter. The fluorescence signal associated with IgG’s presence was very weak in the non-injured spinal cord with IgG injection (Figure 1A, right panel). This is supported with confocal microscopy (Figure 1B). Images were acquired using a 60X objective at approximately 1000 μm caudal to the injury site. At 24 h after injection, IgG was found outside the vasculature (marked by RECA-1 positive staining) in the spinal cords of injured animals. In contrast, non-injured animals with IgG injection contained no parenchymal IgG (Figure 1B, right panel). This suggests that SCI enabled IgG to cross the blood-spinal cord barrier (BSCB).

Microglia and astrocytes are two important CNS cell types involved in the orchestration of the inflammatory response [37,38]. Having demonstrated that IgG appears to cross the BSCB at 24 h after SCI, we assessed whether IgG would co-localize with microglia and astrocytes using confocal microscopy. Iba-1 is a marker for microglia/macrophages. Since monocytes do not infiltrate the injured spinal cord until 2 to 3 days after SCI, most of the Iba-1 positive cells are likely microglia. IgG (red), although in the same vicinity, did not co-localize with Iba-1 (green) positive cells in IgG-injected animals (Figure 1C). Importantly, IgG was not observed in saline injected animals even though the secondary antibody specific to human IgG was applied to these sections (Figure 1C, lower panel).

Astrocytes were stained with anti-glial fibrillary acid protein (GFAP). We observed co-localization between GFAP (green) and IgG (red) in animals with IgG treatment (Figure 1D). Again, there was no IgG signal in saline injected animals, as expected (Figure 1D, bottom panel). This observation suggests potential interaction between IgG and astrocytes in IgG-treated animals.

#### Intravenous IgG significantly reduces neutrophil infiltration

**Figure 2:**
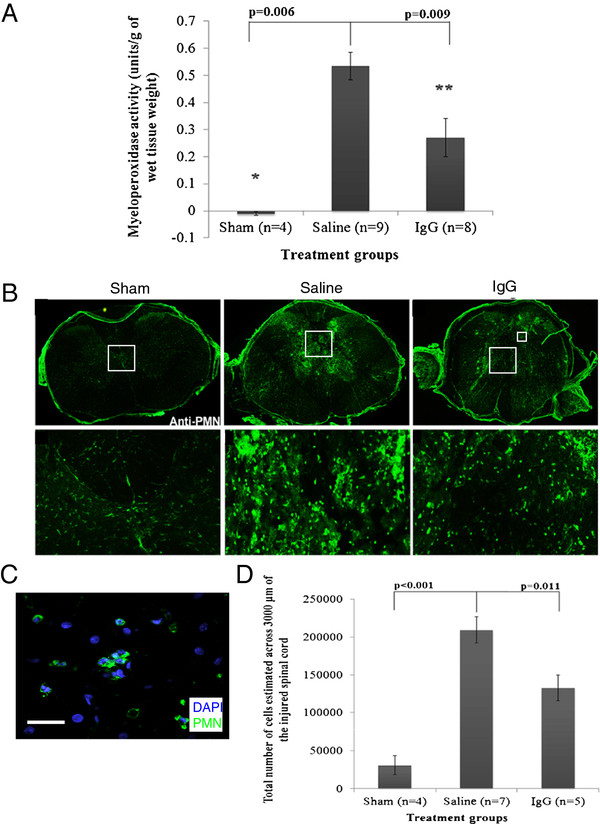
**IgG reduces neutrophil extravasation.** (**A**) Myeloperoxidase (MPO) activity was detected in sham (*n* = 4), intravenous saline (saline, *n* = 9), and intravenous IgG (IgG, *n* = 8) groups. MPO was used to indirectly measure the presence of neutrophils 24 h after SCI. IgG-treatment significantly reduced MPO activity relative to saline control (ANOVA *P* <0.05, Bonferroni post-hoc test *P* = 0.009). (**B**) Representative fluorescence images demonstrate neutrophil distribution at the injury epicenter in the spinal cord at 24 h post SCI (PMN; green). Neutrophils were observed in both saline and IgG-treated animals. (**C**) A representative confocal image of neutrophils (PMN; green) and DAPI (blue) was taken from the indicated area (smaller box), and the scale bar represents 75 μm in length. (**D**) Stereological cell counts demonstrated significantly fewer neutrophils in IgG-treated animals relative to saline control injected animals across 3,000 μm of the injured spinal cord (one-way ANOVA *P* <0.001; Bonferroni post-hoc test *P* = 0.011). Error bars represent SEM.

Neutrophils (which produce MPO) represent an important component of the innate immune response and infiltrate the injured spinal cord maximally at 24 h after SCI [7]. At 24 h after SCI, MPO activity was found to have significantly increased in animals with SCI compared to sham animals (Figure 2A). The MPO activity in the injured spinal cord of IgG-treated animals was significantly lower than the MPO activity in saline-treated animals (one-way ANOVA *P* <0.001; Bonferroni post-hoc test *P* = 0.009). 

Next, we used immunohistochemistry and stereology to quantify the number of neutrophils in the injured spinal cord at 24 h after SCI. Neutrophils were observed in both saline and IgG-treated SCI animals (Figure 2B middle and right panels) but not in sham animals (Figure 2B, left panel). Figure 2D demonstrates that significantly fewer neutrophils were counted in IgG-treated animals compared to saline controls (one-way ANOVA *P* < 0.001; Bonferroni post-hoc test *P* = 0.011).

#### Intravenous IgG significantly reduces MMP-9 expression

**Figure 3:**
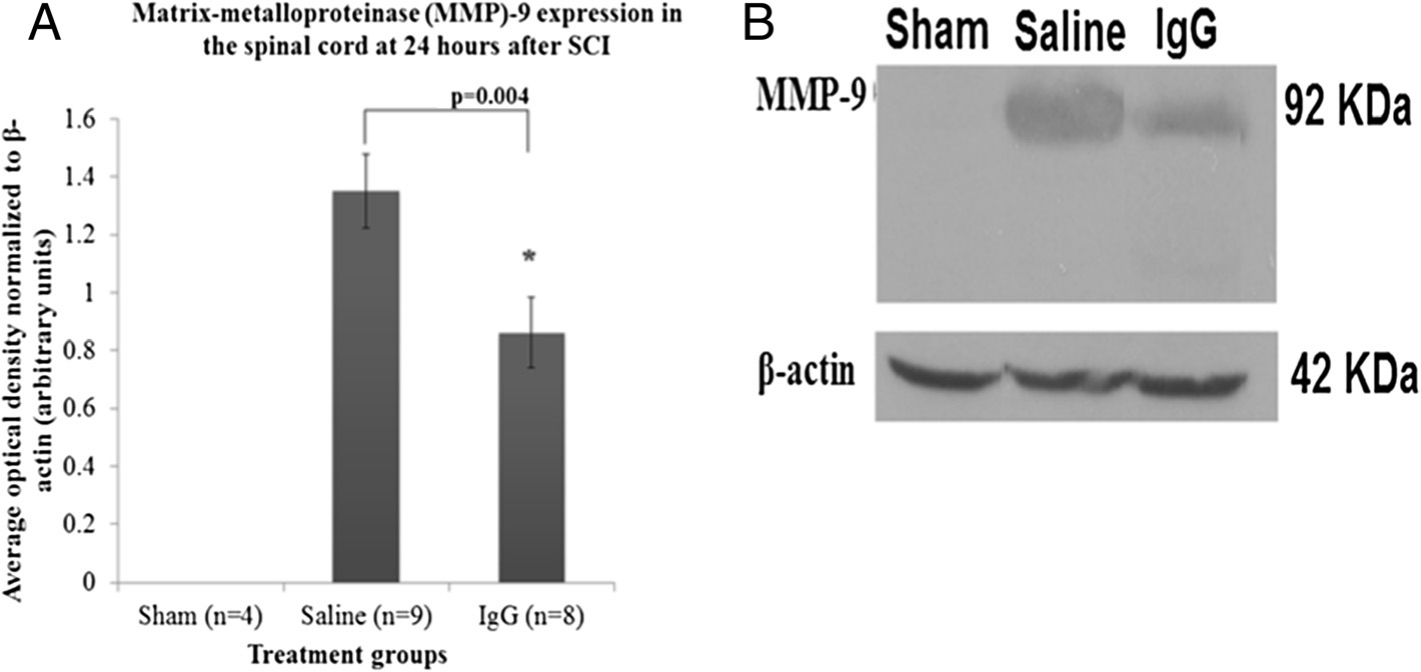
**IgG reduces MMP-9 expression.** (**A**) Matrix metalloproteinase-9 (MMP-9) expression was determined by western blot analysis at 24 h after SCI. Three treatment groups were studied including laminectomy only (sham, *n* = 4), intravenous saline (saline, *n* = 9), and intravenous IgG (IgG, *n* = 8). IgG treatment was associated with a significant reduction in MMP-9 level in the injured spinal cord (*P* = 0.004). Error bars represent SEM. (**B**) A representative western-blot of MMP-9 is shown. β-actin was used as a loading control.

MMP-9 is a matrix enzyme that is produced by neutrophils and microglia. During inflammation, MMP-9 helps neutrophils cross the endothelial wall to the injury site by degrading the collagen matrix of the basoendothelial wall (Delclaux, 1996). At 24 h after SCI, we observed that the level of MMP-9 increased significantly in the injured spinal cord of saline-treated animals (Figure 3 A and B). However, MMP-9 expression was not detected using western blot in sham animals. Densitometric and statistical analysis showed that IgG treatment significantly reduced MMP-9 expression compared to the saline controls (*t*-test *P* = 0.004).

#### Intravenous IgG treatment significantly reduces pro-inflammatory cytokine and chemokine levels in the injured spinal cord

**Figure 4:**
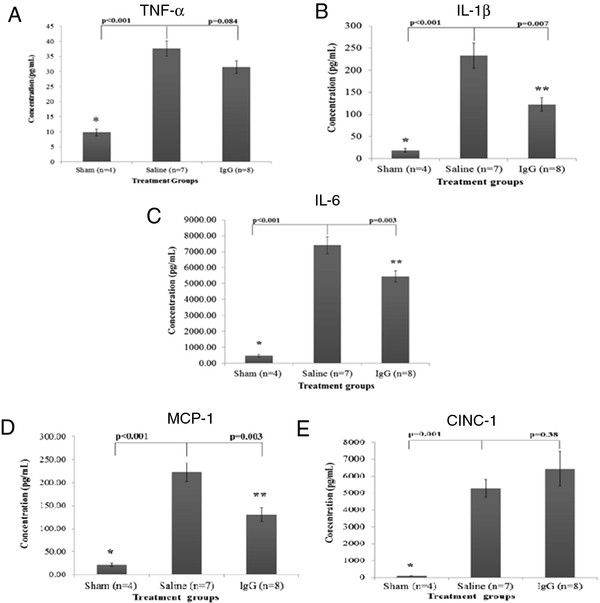
**IgG reduces IL-1β, IL-6, and MCP-1 expression.** Expression of cytokines (TNF-α, IL-1β and IL-6) and chemokines (MCP-1 and CINC-1) at 4 h after SCI was determined with ELISA in the spinal cord homogenates of sham, saline, and IgG-treated animals. SCI resulted in a significant increase of all inflammatory mediators relative to non-injured sham animals. (**A**) There was no difference in TNF-α protein expression between saline and IgG-treated animals (*P* = 0.084). (**B**) IgG treatment was associated with a significant reduction in IL-1β following SCI in rats (*P* = 0.007). (**C**) IL-6 protein expression was significantly reduced in IgG-treated animals relative to saline controls (*P* = 0.003). (**D**) IgG treatment was associated with a significant decrease in MCP-1 protein expression relative to saline controls (*P* = 0.003). (E) IgG treatment had no effect on the level of CINC-1 after SCI relative to saline control injected animals (*P* = 0.38). Error bars represent SEM.

Pro-inflammatory cytokines such as TNF-α, IL-1β, and IL-6 have been shown to mediate the inflammatory response in many conditions including SCI. Using multiplex ELISA, we examined the expression of cytokines and chemokines in spinal cord homogenates at 4 h following injury. Expression of these inflammatory mediators was significantly higher in injured animals compared to sham animals. Figure 4A demonstrates that although TNF-α levels in IgG-treated animals were lower than in saline-treated animals, the difference was not statistically significant (one-way ANOVA *P* <0.01; Bonferroni post-hoc test *P* = 0.084). However, IL-1β (Figure 4B) and IL-6 (Figure 4C) levels in IgG-treated animals were significantly lower than in saline control animals. These data suggest that IgG treatment is associated with significant reductions in the level of IL-1β (one-way ANOVA *P* <0.001; Bonferroni post-hoc test *P* = 0.007) and IL-6 (one-way ANOVA *P* <0.001; Bonferroni post-hoc test *P* = 0.003) in the injured spinal cord at 4 h after SCI.

Since IgG treatment is associated with significant reductions in MPO activity, IL-1β, and IL-6 levels, we examined whether IgG had any effect on the level of neutrophil and macrophage chemoattractants - CINC-1 (Figure 4D) and MCP-1 (Figure 4E), respectively. SCI increased the expression of these inflammatory mediators relative to sham animals. IgG treatment significantly reduced MCP-1 protein levels at 4 h after SCI relative to saline controls (one-way ANOVA *P* <0.001; Bonferroni post-hoc test *P* = 0.03). However, IgG did not significantly reduce the expression of CINC-1 compared to saline controls (one-way ANOVA *P* <0.001; Bonferroni post-hoc test *P* = 0.38).

#### Intravenous treatment with IgG significantly reduces scar and cavitation in the injured spinal cord and enhances preserved neural tissue

**Figure 5:**
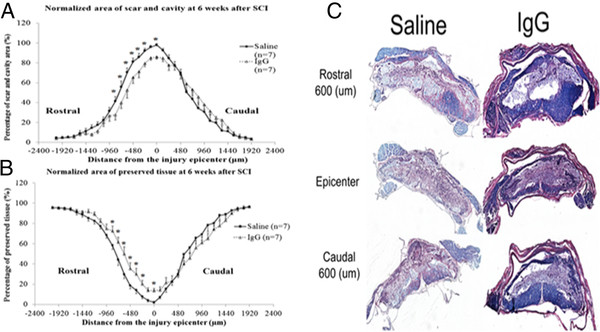
**IgG reduces scar and cavity formation and preserves neural tissue.** (**A**) The distribution of scar and cavity area over 4000 μm of the injured spinal cords of sham (*n* = 4), saline (*n* = 7), and IgG (*n* = 7) animals was calculated at 6 weeks following injury. There was a treatment effect in the area of cavitation and scar in IgG-treated animals (two-way ANOVA, *P* = 0.013). IgG-treated animals had significantly less scar and cavity area at 600 μm (*P* = 0.012) and 720 μm (*P* = 0.042). (**B**) Preserved tissue (remaining grey and white matter) in injured spinal cords was calculated. There was an overall treatment effect in the amount of tissue sparing in IgG-treated animals (two-way ANOVA, *P* = 0.015). IgG-treated animals had significantly more preserved tissue compared to saline-treated animals at a distance of 600 μm (*P* = 0.002), 720 μm (*P* = 0.017), and 840 μm (*P* = 0.044) rostral to the injury epicenter. (**C**) Representative H&E/LFB images are shown. Error bars represent SEM.

In order to determine whether attenuation of the inflammatory response at 4 h and 24 h after SCI affects the amount of tissue sparing and extent of scarring and cavitation, we performed spinal cord histological analyses at 6 weeks post injury. The injury epicenter of the injured spinal cords had the largest area of scar and cavity and the smallest area of tissue sparing (Figure 5 A and B). Representative H&E/LFB images are shown in Figure 5C. As the distance from the injury epicenter increased, the area of scar and cavity decreased and the area of preserved tissue increased. There was an overall treatment effect in the scar/cavitation size and amount of tissue sparing in the IgG-treated animals (two-way ANOVA *P* = 0.013 for each). IgG-treated animals had significantly less scarring at the injury epicenter (0 μm) and at distances 240 μm, 360 μm, 480 μm, 600 μm, 720 μm, and 840 μm rostral to the injury epicenter (Bonferroni post-hoc test *P* < 0.05 for each distance; Figure 5A). At 6 weeks after SCI, IgG-treated animals had significantly more preserved tissue compared to the saline-treated animals at the injury epicenter (0 μm) and at distances 240 μm, 360 μm, 480 μm, 600 μm, 720 μm, and 840 μm rostral to the injury epicenter (Bonferroni post-hoc test *P* <0.05 for each distance; Figure 5B).

#### Intravenous IgG treatment increases neurobehavioral recovery after spinal cord injury

**Figure 6:**
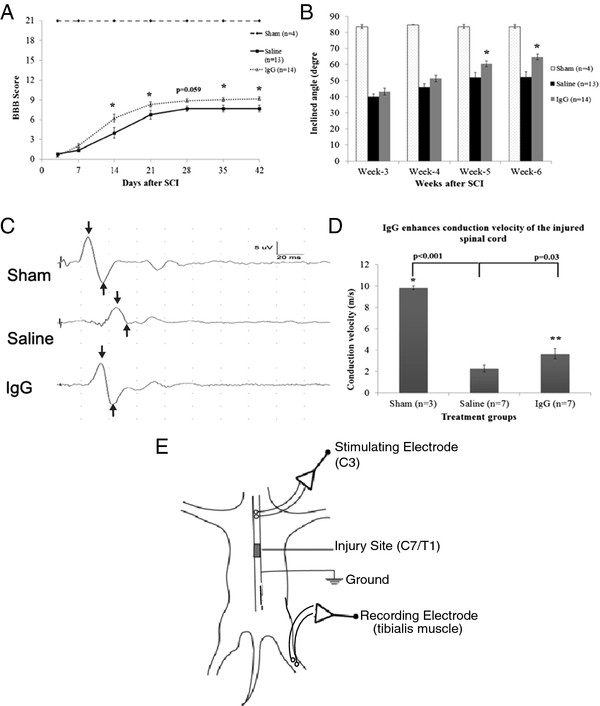
**IgG increases neurobehavioral recovery and axonal function.** (**A**) Hind-limb functional recovery was assessed weekly for 6 weeks following SCI. Scores were assigned according to the Basso Beattie Bresnahan (BBB) Scale. Sham animals (*n* = 4) displayed no hind-limb functional deficits and were given a score of 21. IgG treatment was associated with significant improvement in hind-limb functional recovery (two-way ANOVA *P* <0.001). The differences in BBB scores between IgG- and saline-treated animals were statistically significant at weeks 2, 3, 5, and 6 (Bonferroni post-hoc test *P* <0.001, *P* = 0.015, *P* = 0.031, and *P* = 0.017, respectively). (**B**) The inclined plane test was used to assess functional recovery of the saline (*n* = 13) and IgG (*n* = 14) groups. The inclined plane test measures the hind- and forelimb strength and coordination necessary to maintain a horizontal position on an inclined plane. Larger inclined angles are associated with better functional recovery. At weeks 5 and 6, IgG-treated animals performed significantly better than saline-treated animals on the inclined plane test (two-way ANOVA *P* <0.001; Bonferroni post-hoc test week 5 *P* = 0.012 and week 6 *P* <0.001). (**C**) Motor-evoked potentials (MEPs) were recorded in injured spinal cords at 6 weeks following SCI. Conduction velocity in both the IgG and vehicle control group was significantly reduced compared to uninjured sham rats (one-way ANOVA *P* <0.05; Bonferroni post-hoc test *P* <0.01). IgG-treated animals demonstrated significantly faster conduction velocities compared to saline control animals (*P* = 0.03). (**E**) Schematic diagram showing how MEPs was recorded in the injured spinal cords at 6 weeks following SCI.

Functional recovery, as tested by the BBB (Figure 6A) and the inclined plane test (Figure 6B), was significantly greater in IgG-treated animals compared to the saline control group. Sham animals displayed no functional deficits throughout the 6-week period and thus scored 21 on the BBB scale. The differences in BBB scores between IgG- and saline-treated animals were statistically significant at weeks 2, 3, 5, and 6 (two-way ANOVA *P* <0.001; Bonferroni post-hoc test *P* <0.001, *P* = 0.015, *P* = 0.031, and *P* = 0.017, respectively). The BBB results suggest that IgG treatment is associated with increased hind-limb functional recovery compared to saline controls. More specifically, animals in the IgG-treated group on average had extensive movement in all three joints and some level of weight support, while saline-treated animals on average had extensive movement in all three joints without any weight support. IgG-treated animals also displayed a greater recovery than saline-treated animals in the inclined plane test from week 3 to week 6 (two-way ANOVA *P* <0.001; Bonferroni post-hoc test week 5 *P* = 0.012 and week 6 *P* <0.001). To support the neurobehavioral improvement, motor-evoked potentials were recorded in injured spinal cords at 6 weeks following SCI (Figure 6C). Conduction velocity in both the IgG and vehicle control group was significantly reduced compared to uninjured sham rats (one-way ANOVA *P* <0.05; Bonferroni post-hoc test *P* <0.01). IgG-treated animals had significantly faster conduction velocity compared to saline control animals (*P* = 0.03). Representative MEP tracings for each group are shown.

## Discussion

In this study, we present a novel application of IgG in attenuating the effects of inflammation-mediated damage and improving neurobehavioral recovery after cervical SCI. More specifically, IgG-treatment is associated with a significant reduction in the level of pro-inflammatory cytokines and chemokines (IL-1β, IL-6, and MCP-1), in the level of cytotoxic enzymes (MMP-9 and MPO), and the level of neutrophil infiltration. Our results are consistent with reported findings from other laboratories which have indicated that pro-inflammatory cytokines and neutrophils can potentiate the initial injury following SCI [20,39,40]. Although the potential use of IgG for traumatic SCI was previously reported by Gok *et al*. [28], Gok and colleagues did not report the neuroprotective mechanism and any long-term neurobehavioral benefit of IgG treatment. Our current manuscript suggests a potential immuno-modulatory mechanism of IgG and provides a more detailed demonstration of the immuno-modulatory effects of IgG at the molecular, cellular, and neurobehavioral level.

### IgG treatment significantly reduces the level of neutrophil infiltration and cytotoxic enzymes in the injured spinal cord

Neutrophils, which represent an important component of the innate immune response, infiltrate the spinal cord within hours of injury, and maximal cell numbers are reached at 24 h after SCI [7]. Neutrophils can increase the extent of tissue injury after SCI by producing reactive oxygen species (ROS) and reactive nitrogen species (RNS) [41-43] that can damage proteins, DNA, and lipids. Neutrophils can further increase the extent of the inflammatory response by producing pro-inflammatory mediators such as TNF-α, IL-1β, and IL-8 [44]. MMP-9 secreted by neutrophils can degrade the collagen matrix of the blood-spinal cord barrier and increase leukocyte infiltration [45,46]. Blockade of neutrophil infiltration using various strategies has been demonstrated to reduce oxidative damage and to improve neurobehavioral recovery in several rat models of SCI [17,18,39,40]. In the current study, we found that intravenous IgG treatment was associated with a significant reduction in MPO activity at 24 h after SCI (Figure 2A). MPO is an enzyme produced by neutrophils and stored in the azurophilic granules, and as such, MPO activity was used as an indirect measure of neutrophil presence in the injured spinal cord [47]. Our finding is consistent with a previous report by Gok *et al*., in which IgG was associated with a significant reduction in MPO activity when it was injected intraperitoneally immediately following SCI [28]. We also observed a significant reduction in neutrophil infiltration to support the MPO data (Figure 2B and C).

### IgG treatment significantly reduces pro-inflammatory cytokine and chemokine levels in the injured spinal cord

Immediately after SCI, a rapid increase in the levels of TNF-α, IL-1β, and IL-6 is observed in the spinal cords of humans and animal models of SCI [7,48-50]. It has been suggested that these cytokines exacerbate the initial damage and contribute to neurodegeneration following CNS injury [51]. Following CNS injury, pro-inflammatory cytokines play an important role in activating and recruiting leukocytes including neutrophils, monocytes, and lymphocytes. Pro-inflammatory cytokines promote leukocyte infiltration to the site of injury by increasing the expression of vascular cell adhesion molecules on the surface of endothelial cells [52,53] and by regulating the expression and secretion of chemokines [38,54,55]. IL-6 has previously been shown to regulate the production of IL-8 and MCP-1 expression in a mouse model of skin pouch inflammation [54]. Furthermore, IL-1β has previously been shown to regulate the production of CINC-1 and MCP-1 in a mouse model of SCI via the IL-1R/MyD88 axis, and astrocytes were shown to be the primary cellular source of the aforementioned cytokines [38].

IgG treatment was associated with a significant reduction in IL-1β and IL-6 levels in the injured spinal cord. This finding may explain why neutrophil infiltration is attenuated upon intravenous administration of IgG after SCI. In fact, previous studies in SCI have shown that reductions in the expression level or disruption in the signaling pathway of either TNF-α, IL-1β, or IL-6 is associated with a significant reduction in neutrophil infiltration in SCI models as well as other models of acute inflammation [38,54,56,57]. Along the same lines, if TNF-α or IL-1β is injected into a non-injured spinal cord or IL-6 is over-expressed in an injured spinal cord, then leukocyte infiltration has been shown to be enhanced [58-60]. Our findings suggest the observed reduction in neutrophil infiltration may be due to IgG’s mechanism of action in reducing the level of pro-inflammatory cytokines in SCI. In our future experiments, using our established experimental paradigms, we could employ antibodies to block Fc receptors as an approach to elucidating the immuno-modulatory mechanism of IgG.

We observed that IgG treatment was associated with a significant reduction in MCP-1 levels but not CINC-1. In addition to astrocytes, endothelial cells have been shown to co-localize with CINC-1 after SCI in the perivascular region [18]. Currently, there is insufficient scientific evidence describing the primary cellular source of these chemokines and the signal transduction pathways that regulate the production of these chemokines. The lack of strong supporting evidence in the literature presents major challenges in outlining the relationship between IL-1β, IL-6, MCP-1, and CINC-1 in animal models of SCI. The heterogeneity in species and injury mechanisms of animal models of SCI make it difficult to draw correlations between published findings and the observations stemming from the present body of work. Future experiments examining the cellular targets of IgG and the cellular sources of cytokines and chemokines in rats will illuminate the complex interaction and regulation of cytokine and chemokine expression.

Although qualitative in nature, our immunohistochemistry experiments using astrocyte and microglia as specific markers provide valuable insights into the cellular target(s) of IgG in SCI. We observed that IgG only co-localized with astrocytes and is associated with significant reduction in the level of IL-1β and IL-6. Previous studies on cellular sources of pro-inflammatory cytokines might explain why IgG had only a modest attenuation in the level of TNF-α after SCI. Microglia/macrophages and astrocytes are the major cellular sources of TNF-α, IL-1β, and IL-6 immediately after SCI [37]. The level of IL-1β but not TNF-α is maintained by neutrophils and astrocytes at later time points after SCI [37,48]. Although determining the exact cellular target and the underlying mechanism of IgG is beyond the scope of this study, the selective reduction of cytokine expression and the specific astrocytic co-localization of IgG suggest some degree of cellular specificity in IgG’s mechanism of action. Although we did not observe co-localizations between IgG and Iba-1 positive cells in this study, we cannot rule out the potential interaction between microglia and IgG in the acute phase as well as the sub-acute phase of SCI. The underlying mechanism and the molecular target of IgG on astrocytes and/or microglia can be examined in future studies.

### IgG treatment significantly increases tissue preservation and ameliorates neurobehavioral recovery

Previous studies have reported that attenuation of IL-6 signaling [61,62], blocking neutrophil infiltration [15,16,39,40,63], or reducing MMP-9 expression [46] separately after SCI results in tissue preservation and improvement in hind-limb locomotor function. In this study, we show that IgG treatment is associated with a decrease in the area of scar and cavity (Figure 5A), an increase in tissue sparing (Figure 5B) and a significant improvement in neurobehavioral recovery (Figure 6A and B). Importantly, spinal cord axonal conduction changes (Figure 6C) were in agreement with our neurobehavioral outcomes. The results suggest that axonal conduction is severely impaired following injury and IgG treatment provides some degree of axonal preservation or improvement in axonal signaling. This is in agreement with the histological, molecular and behavioral data. Although the magnitude of tissue preservation in the IgG-treated animals may appear modest compared to the saline control group, it has previously been demonstrated that only 10% of axons are needed to achieve significant functional recovery after SCI [30]. Our tissue preservation and neurobehavioral findings are consistent with findings by Gris and colleagues [15], in which they attenuated leukocyte infiltration with anti-CD11d antibodies and observed modest levels of tissue sparing and a two-point improvement in the BBB score after moderate to severe SCI. In conjunction with other groups’ findings, our data reinforce the detrimental role of the acute inflammatory response in SCI. Our results also support the underlying rationale of immuno-modulatory therapy in attenuating the effects of the acute inflammatory response after SCI.

## Conclusion

Although a short treatment window of 15 min was employed in our current study, we have demonstrated the novel use of IgG in modulating neuroinflammation and ameliorating neurobehavioral recovery after cervical SCI in rats. The findings from this study also reinforce the finding of a detrimental role for the acute inflammatory response. IgG represents a promising immuno-modulatory agent that reduces inflammation-mediated damage. IgG is a cost-effective and easily deliverable therapeutic that could be used in the acute stage of a combinatorial treatment strategy. Considering there is currently no effective treatment option for SCI, these findings could have a significant impact on the treatment of SCI. Before this can be realized, supporting evidence and optimization of efficacy are required. Future studies will examine a more clinically relevant delayed time of IgG delivery and establish a dose response.

## Competing interests

The authors declare that they have no competing interests.

## Authors’ contributions

DHN participated in the design of the study, carried out the surgery, actively involved in all experimental aspects of the study, coordinated the experiments, and drafted the manuscript. NC helped with editing the manuscript and performing biochemical assays, histological and immunohistochemical analyses, and neurobehavioral assessment. KS carried out the electrophysiology study and helped draft the manuscript. JWA assisted with ELISA analysis, neurobehavioral assessment, and manuscript compilation. JW administered treatments, assisted neurobehavioral assessment, and carried out immunohistochemical staining and confocal microscopy imaging. MGF conceived the study, participated in its design, and helped draft the manuscript. All authors read and approve the manuscript.
